# Traditional beliefs and practices in the postpartum period in Fujian Province, China: a qualitative study

**DOI:** 10.1186/1471-2393-7-8

**Published:** 2007-06-21

**Authors:** Joanna H Raven, Qiyan Chen, Rachel J Tolhurst, Paul Garner

**Affiliations:** 1 International Health Group, Liverpool School of Tropical Medicine, Pembroke Place, Liverpool, UK; 2 Health Care Department, Fujian Provincial Maternity and Children's Hospital, Fuzhou, Fujian Province, China

## Abstract

**Background:**

*Zuo yuezi *is the month postpartum in China associated with a variety of traditional beliefs and practices. We explored the current status of *zuo yuezi *from social, cultural and western medical perspectives.

**Methods:**

We interviewed family members (36) and health workers (8) in Fujian Province, selecting one rural and one rapidly developing urban county. We asked about their traditional beliefs and their behaviour postpartum. We used a framework approach to identify main themes. We categorised reported behaviour against their probable effects on health, drawing on Western standards.

**Results:**

Respondents reported that *zuo yuezi *was commonly practiced in urban and rural families to help the mother regain her strength and protect her future health. *Zuo yuezi *included: dietary precautions, such as eating more food and avoiding cold food; behavioural precautions, such as staying inside the home, avoiding housework and limiting visitors; hygiene precautions, such as restricting bathing and dental hygiene; and practices associated with infant feeding, including supplementary feeding and giving honeysuckle herb to the infant. Respondents reported that the main reasons for adhering to these practices were respect for tradition, and following the advice of elders. Categorised against Western medical standards, several *zuo yuezi *practices are beneficial, including eating more, eating protein rich food, avoiding housework, and daily vulval and perineal hygiene. A few are potentially harmful, including giving honeysuckle herb, and avoiding dental hygiene. Some women reported giving infants supplementary feeds, although *zuo yuezi *emphasises breast feeding.

**Conclusion:**

*Zuo yuezi *is an important ritual in Fujian. In medical terms, most practices are beneficial, and could be used by health staff to promote health in this period. Further research on reported potentially harmful practices, such as supplements to breast feeding, is needed.

## Background

Childbirth is a time of transition and social celebration in many societies, signalling an adjustment of cultural responsibilities [1]. Women's progression from birth to childrearing is influenced by economy, religion, kinship system and the growing sophistication of communications and medical technology [2]. In some societies, there is a continuum between traditional and modern care, with some households operating at the traditional end, others at the modern end, with the majority somewhere in between [3]. Internationally, many studies describe the traditional beliefs and practices surrounding childbearing [for example, [4-8]]. Some traditional practices are beneficial to the mother and baby, whereas other practices are not.

In China, the one month postpartum is called *zuo yuezi*. The literal translation means "doing the month". Traditionally, a woman remains at home during this period. During this time, her behaviour in relation to diet, activity and hygiene is determined by tradition, and the theory behind traditional Chinese medicine (TCM) underlies some of these beliefs and practices [9,10]. Health is seen as harmony between *yin qi *and *yang qi; *and illness an imbalance between the two forces [10]. Pregnancy is a *yang *state, but during childbirth the woman loses heat and becomes *yin*. The behaviour around diet, activity and hygiene that comprise "doing the month" is to restore the equilibrium [2,9,11,12].

Other research examines how *zuo yuezi *is adapted in Chinese women migrants to a Western society [4,13,14]; others describe the postpartum period in China and Hong Kong, with little evaluation of what the practices mean in relation to health [9,11]. One study analysed the practices from the perspectives of folk, Chinese and western medicine beliefs [12].

We were interested in the effects of the traditional practices on health and how health staff should view these practices as beneficial, irrelevant or harmful, to help guide how they might modify women's behaviour at this time. In our study, we aimed to examine reported current behaviour from the perspectives of families, health workers and traditional medicine practitioners, aware that practices may be changing as a result of the recent rapid economic and social development. We then assessed the potential effects of the practices on the health of mother and baby from western health perspective.

## Methods

### Setting

This study was carried out at one urban and one rural site between May and November 2004: the urban centre of Fuzhou, and Fuqing County in Fujian Province. National economic reform since 1978 and rapid economic and social development has led to increases in the gross domestic product in Fujian from 8 billion Yuan in 1980 to 576 billion Yuan in 2004 [15]. In Fujian, this has had an impact on health and social well being of the province population. Average life expectancy has risen from 35 years in 1950 to 74 years in 2001 and illiteracy rate has decreased from 16% in 1990 to 7% in 2000 [15,16]. Fuzhou is the capital city of the province and has a total population of 5.7 million. Its main sources of employment are industries and private commerce. Fuqing County, located approximately 100 km from Fuzhou has a population of 1.2 million dispersed through 21 towns and 467 villages. The main sources of income are small businesses, farming and overseas employment. Fujian province is one of the leading emigrant sending provinces in China and in 2000, 25% of Fujian province emigrants came from Fuqing County [17].

### Participants

Study participants were families, health workers and traditional medicine practitioners from both areas. The participant selection criteria and rationale, methods of data collection and topics discussed are summarised in table 1.

**Table 1:** Summary of selection process and interview topics

**Method**	**Participant**	**Selection Criteria and Rationale**	**Topics discussed**
Semi- structured interviews	Families: 12 mothers 12 husbands 12 grand-mothers^#^	Mother gave birth to living baby in last 4 months, but was not currently in postpartum period*. Grandmother cared for daughter during the postpartum period. Range of ages in mother.	Practices, beliefs and reasons. Extent to which these practices are implemented. Perceptions of practices, their benefits and harms. Decision making processes. Health problems related to practices.
Key informant interviews	4 Health workers	Provided postpartum services in study settings. Experience of practices.	Practices being implemented. Health problems related to practices.
Key informant interviews	4 Traditional medicine practitioners	Worked in Maternal and Child Health Care in study settings	Reasons for practices. TCM theory in relation to practices.

*Interviewing mothers during the postpartum period was felt to be intrusive. Families would still have good recall of events of the postpartum period during the 4 months.

^# ^Grandmother refers to infant's grandmother.

In Fuzhou, doctors from Fujian Provincial Maternal and Children's Hospital recruited six families. In Fuqing County, six families were recruited through Fuqing County Maternal and Child Health Hospital and village health centres. We identified health workers for interview through hospitals and health centres where families received care. Traditional medicine practitioners were chosen from Fujian Provincial Maternal and Children's Hospital and from Fuqing Traditional Medicine Hospital. We purposively selected interviewees to help explore the key topics being studied [18]. We aimed to arrive at the sample size following the principle of saturation whereby interviews should continue until no new data are generated [18]. After completing the interviews with twelve families, saturation was achieved with regard to key topics.

### Data collection

Semi structured interviews and key informant interviews were used to uncover the interviewee's own framework of meanings. These interviews can provide rich data, which can be used to describe and explain people's behaviour in relation to their social and cultural context [19]. The interviewer used topic guides covering the following aspects: diet, hygiene, rest and activity, and infant feeding. We also explored beliefs about practices, perceptions of their benefits and harms, effects of these practices, and decision making processes in families (table 1).

Interviews were conducted in Chinese. They lasted between 40 and 80 minutes. All interviews were tape recorded with interviewees' permission, for transcription and analysis. Interviews with family members took place in family homes. Interviews with health workers and traditional medicine practitioners were carried out in private rooms of Fujian Provincial Maternity and Children's Hospital and Fuqing County Maternal and Child Health Hospital. All locations were acceptable to the interviewees.

The interviews were transcribed in Chinese. To minimise data loss interviews were then jointly translated and transcribed by the main author and the translator, and a member of the research team (QC) checked the translated transcripts with the Chinese transcripts.

### Analysis

Data were analysed using a framework approach. The thematic framework is used to classify and organise data according to key themes, concepts and emergent categories. It facilitates rigorous and transparent analysis [20]. Transcripts were read and re-read separately by the investigators to identify emerging themes. A coding framework was devised based on these themes and all data were coded with this framework. A chart was created for each theme, using coded segments of data. These charts were used to describe similar and divergent beliefs and practices, develop explanations and find associations between them. Triangulation of research participants and researchers enabled crosschecking of the data and brought out different points of view.

### Ethical considerations

The Ethics Committee of Liverpool School of Tropical Medicine and Fujian Provincial Health Bureau granted approval for the study. Ethical procedures were followed according to the Human Research Ethics Committee approval.

## Results

Socio-demographic characteristics of the families and health workers and traditional medicine practitioners are presented in table 2. All health workers and traditional medicine practitioners interviewed were female.

**Table 2:** Demographic characteristics of families, health workers and traditional medicine practitioners

**Characteristics**	**Mother**	**Grand mother**	**Husband**	**Health worker**	**Traditional medicine practitioner**
**Age:**					
20–30	11		6	2	1
31–40	1		6	1	1
41–50		2		1	2
>51		10			
**Occupation:**					
Home duties	5	5			
Farmer		4	2		
Self employed	2		3		
Government employee	5	2	1		
Private employee		1	6		
**Experience of maternal health:**					
<1 year					
1–3 years				1	1
4–7 years				1	
>8 years				2	3

The following section illustrates five major themes that emerged from interviews: reasons for "doing the month", dietary precautions, hygiene, behavioural precautions and infant feeding.

### Tradition versus change: reasons for "doing the month"

All families followed an adapted version of the ritual of "doing the month". The purpose is to help the new mother regain her strength and health in order to care for the new baby, resume normal activities and protect her future health. In most families this lasted thirty days. Traditionally, the grandmother usually looks after the mother. There is widespread emphasis that "doing the month" properly will safeguard future health. It was commonly believed that this period is the weakest time of a woman's life. As one husband explained:

"Some women have good health, but they do not do the postpartum period well, and their health becomes poor. Other women with poor health do the postpartum period well and their health becomes better. It is a period of great change, so we do not want to take any risk" (Husband, urban family 3).

However, conflict of knowledge within families was apparent. Some information was obtained from books, Internet and health professionals, although most came from older family members. One husband described this:

"Old family members and friends tell us to have traditional food during this period. We follow their advice because we don't know what to do in this period. But if we do follow this diet we still don't know if we will have some problems. The doctor gave us some suggestions, but our parents promoted the traditional way. It is difficult to make a choice" (Husband, urban family 4).

Most families wanted to follow the beliefs and practices of their parents and grandparents. Some perceived modern beliefs and practices as "foreign" to their cultural context. They were reluctant to risk following a modern practice and a problem occurring.

"We are not certain if the foreign way can be done here. It is not that we don't believe in it, we just don't want to try that way. So if we can follow the traditional way, we just follow it" (Husband, urban family 2).

Health workers spoke of changes in the traditional practices that have occurred over the years and reasons for these changes. They cited social and economic development, accompanied by improvements in education, communication and living standards. One health worker explained:

"I think our society has developed and the minds of people have changed. It has changed naturally... living standards have improved; there is a better and more hygienic environment. The buildings are brighter and we have more space" (Health worker 1, rural).

### Dietary precautions

Families' explanations revealed several dietary precautions that were taken seriously during the postpartum period.

#### Eating more food

There was a belief that the postpartum woman should eat a lot of food. Two main reasons were given: first, women at this time are weak, and food will help rebuild her strength, promote recovery and improve breastfeeding. Second, the carer's own experience (usually the mother's own mother) of "doing the month" was, in some instances, during a time when food was short. They believed this affected their health long term. As one carer explained:

"I hope she can eat as much as possible. When I did the month I only ate three meals a day. I hope she can eat more to help her recover more quickly" (Grandmother, rural family 4).

Mothers reported that they consumed more food than normal. The number of meals ranged from five to eight in a day, starting at 5 am and finishing with a meal before sleeping at night. However, this was not true for all mothers: some were not able to eat so much food. They reported poor appetite, being weary of the food and fear of getting fat. For some women, there was a conflict between limiting the types of food that could be eaten and increasing the amount of food. One mother explained problems with this diet:

"After one month of eating the same food, I was bored and fed up with eating" (Mother, rural family2).

Health workers and traditional medicine practitioners said that they thought eating more food and a variety of food was generally beneficial, but some women gained too much weight because they did little exercise and ate too much fat in their diet.

#### Eating "hot" food (protein rich)

All families believed in eating food seen as "hot" within the context of TCM. Meat and eggs were regarded as "hot" foods. Food could also be made "warmer" by adding ginger and wine. This food, which was also viewed as full of protein, was thought to enrich the blood, help the mother's recovery, encourage expulsion of lochia and stimulate production of breast milk. Traditional medicine practitioners and health workers all believed this, recognising that having lost energy and blood during delivery, both *yin *and *yang *are weakened. In order to restore the balance, "warm" food should be consumed. For example, one mother said:

"We have to eat more hot food because we bleed at delivery. Hot food will enrich the blood and help the recovery process. Cold food will stop this from happening" (Mother, rural family 4).

All mothers consumed "hot" or "warm" foods during the month. Mothers living in the city ate mostly chicken, and often added wine, ginger or dates. However, some families said food that was too "hot" could cause the baby to become restless, and cause nosebleeds in the mother. They therefore reduced the frequency and amount of wine added to food. In the rural area mothers had a more varied diet: eating a lot of fish, rabbit, pork, chicken and duck, but few added wine to food. This mother illustrates a common reason for following the strict diet, related to the perceived long term benefits:

"My grandmother said to me – 30 days will pass easily, but 30 years will pass with more difficulty. I think the benefits of the diet will last for 30 years" (Mother, urban family 2).

#### Avoiding "cold" food (fruit and vegetables)

Almost all families believed that food viewed as "cold" within TCM should be avoided during this period. Many grandmothers believed most fruit and vegetables to be "cold", and were unable to identify "warmer" ones. Rural families identified numerous effects of eating "cold" food: diarrhoea in baby and mother, body swelling, stomach discomfort, aches and pains and cough. In the urban area they were more concerned that there would be delay in expelling lochia.

"She ate some vegetables, but not very many. They can cause diarrhoea and the baby will also get diarrhoea through the breast milk" (Grandmother, rural family 4).

All mothers in rural and urban areas could identify vegetables and fruit perceived as "warmer" and ate these in the postpartum period in order to improve their own health and enrich the breast milk. However, most mothers ate fewer kinds of vegetables and fruit, avoiding ones thought to be "cold". In the rural area, half the mothers ate vegetables less frequently and in less quantity, whereas the other half said they ate a usual amount of vegetables.

"She eats vegetables – spinach, cauliflower, carrot, fish and meat. Generally she eats the same things as usual, but not so many vegetables. I did not let her eat some cold vegetables, like cabbage. It causes a cough and this can also be passed to the baby through the milk" (Grandmother, rural family 5).

A few mothers complained of mild constipation, which they associated with eating "hot" food and too few vegetables. A few were concerned that lack of vitamins would affect the quality of breast milk. Traditional medicine practitioners and health workers agreed that "cold" food should be avoided. However, they all promoted a diet that included fruit and vegetables. Health workers saw only a few women with constipation, haemorrhoids, anaemia, poor healing or infections.

### Hygiene

Perceptions and practices may be divided into three distinct sub themes: bathing and washing hair; vulval and perineal hygiene; and dental hygiene.

#### No bathing or washing hair

Traditionally, women should not bathe or wash hair in the postpartum period, and this was well known in all families. They believed that as the postpartum woman's skin is loose, water can enter the body through holes in the skin. This will cause body swelling, arthritis and rheumatism later in life or a cold which can be passed to the baby. Similarly, hair washing will cause a headache.

Most urban and rural women stated that they usually bathed or showered daily or alternate days when they were not in the postpartum period. Most rural mothers seemed to adapt the tradition by bathing with boiled water, or boiled water with wine or motherwort herb (a common herb with medicinal properties) to prevent the problems of absorption through the skin. Wine and motherwort are both thought to have disinfecting properties and will therefore prevent infection. They believed that as they were with the baby all the time, they needed to be clean to protect the baby from illness. It also made them feel comfortable and happy.

"I added wine to the water because the skin is loose. It is safer when you add wine to the water. Old people say that you cannot use water to bathe. They say that the skin is loose and the wind can enter through the pores and cause illness" (Mother, rural family 3).

In contrast, most mothers in the urban area used a towel to clean the skin, either dry or dampened with cooled boiled water. A few mothers bathed using boiled water. Health workers said that most women use a towel to clean in this period. Some mothers adopted the more traditional ways as they believed that it was important for their future health. Others simply wished to follow the advice of their elders. This is revealed in one mother's response:

"Old people take care of me. I cannot do everything I like and not listen to them at all. So I also adopted some of their suggestions" (Mother, rural family 2).

Some women recognised that it was only for thirty days and they were able to cope with the discomfort. Others found it more difficult to accept as they were used to bathing frequently.

The health workers found that a small number of mothers developed a skin rash and spots because of restrictions in hygiene. Some mothers were worried that they could not bathe. No other health problems were identified. Traditional medicine practitioners and health workers did not support the traditional practice of not bathing in the postpartum period. They said that there is no harm in bathing with warm water and using motherwort, wine or boiled water is not necessary.

#### Vulval and perineal hygiene

Careful hygiene to reduce the risk of infection seemed to be the more important belief with respect to the vulval and perineal area. All mothers in rural and urban areas washed the vulva area every day. They used alcohol, boiled water or iodine to clean incisions or tears. There were no reported problems with infections or poor healing. Health workers promoted vulval and perineal hygiene. They also stated that most mothers cleaned their incision or tear when they returned home. They reported very few instances of infections or poor healing.

*"When I stayed in the hospital the nurse cleaned the perineum for me. When I came home I used some medicine from the hospital to clean the incision. It healed well. I used boiled water and sometimes iodine to clean it" (Mother, rural family 4)*.

#### No brushing teeth

Many people believed that brushing teeth during this period would make teeth loose and gums bleed. Traditional medicine practitioners and health workers did not agree, saying that it was necessary to brush teeth.

None of the mothers in the rural area and only two in the urban area carried out their usual dental hygiene habits of brushing teeth at least once daily. There were a variety of practices carried out: using cotton to clean teeth for two weeks and then brushing; using a soft brush with hot water to make it softer; swilling the mouth with boiled water for two weeks and then brushing; and swilling the mouth for entire month.

When mothers did not brush their teeth they complained of poor appetite and bad taste or smell in the mouth. Some were only able to carry out the traditional practice for a short time and stressed importance on dental hygiene. The health workers saw no health problems associated with these restrictive practices, except that women complained of a poor appetite.

"In the first 7 days I used cloth to clean my mouth. After 10 days I used a soft brush. I can't avoid brushing my teeth for a whole month. It's impossible" (Mother, rural family 1).

### Behavioural precautions

Staying inside the home, avoiding housework, resting in bed, abstaining from sexual activity and limiting visitors are topics around behaviour which emerged from discussions.

#### Staying inside the home

All families believed that when the mother goes outside wind will enter her body and cause illnesses, namely arthritis and rheumatism later in life but also headache, poor appetite and catching a cold.

"She must avoid wind coming straight on to her body. It can cause arthritis, backache and shoulder ache. This is very important in doing the month" (Grandmother, urban family 4).

This belief was strongly adhered to: all mothers in both urban and rural areas stayed at home. In addition, some urban mothers stayed in their rooms for part of the month, not being allowed into the rest of the flat. Most mothers seemed to be able to cope with this restriction and concentrate on resting and looking after the baby.

"We don't go out of the house because firstly we want to follow tradition and secondly we have to look after the baby and we have no time to go out and enjoy ourselves. It would be better if we did have some time to go out and relax" (Mother, urban family 6).

Although the traditional medicine practitioners supported the practice of mothers staying in the home, they did not give the same rationale. They believed that postpartum women are vulnerable to problems such as increased bleeding and colds, but not to arthritis and rheumatism as suggested by some family members. Most health workers felt that although there was no harm in going outside, there was no need for women to leave the home during this period.

#### Avoiding housework

The belief that a mother should not do housework during the month, as she is weak and needs rest was common. Housework requires her to be in contact with either water or wind, which will then enter the body and cause arthritis and chronic aches.

"I am scared she will get arthritis and backache. I did all the washing when I had my babies and now I have arthritis in my feet" (Grandmother, rural family 2).

In all families there were people to look after the new mothers and do the housework during this period. However, two mothers still did some light housework towards the end of the month. One urban mother said it made her feel less tired and helped time go more quickly. The rural mother did some housework, as the grandmother was busy with farm work. Traditional medicine practitioners and health workers all supported the belief that women should rest during this period.

#### Resting in bed

The traditional belief is that mothers should lie in bed for the whole month, recovering from childbirth and preventing future illnesses by keeping out of the wind. Most of the grandmothers advised this, but the majority of mothers and husbands felt this was not necessary.

"My mother said to rest as much as possible. She suggested lying in bed. She said that too many activities would cause leg pain. So I decided to do some exercise and then rest" (Mother, rural family 4).

Two mothers stayed in bed for the entire month as instructed by the grandmothers. The others wanted to rest, as they were tired from sleep disturbances, and needed time to recover from childbirth.

"During this month, I wanted to sleep a lot because every two hours I had to feed the baby. I had to breastfeed the baby 2 or 3 times a night" (Mother, urban family 3).

Others also wanted to do some walking around the home to promote blood circulation, reduce weight and relieve boredom. Mothers felt uncomfortable, hot and had backache if they stayed in bed for long periods. Only one rural mother and two urban mothers did some other exercises. According to the traditional medicine practitioners, TCM does not support the belief that mothers should stay in bed and not do any activity. Traditional medicine practitioners and health workers both advised some exercises but also plenty of rest during this period, so that the mother would make a good recovery.

#### Abstaining from sexual activity

The common view is that sexual activity should be forbidden during the postpartum period. There were several reasons for this restriction: the woman is weak; she has no energy and is concentrating on looking after the baby; she needs to rest; the scar has not healed; she is still bleeding; and it can cause an infection. Health workers and traditional medicine practitioners supported this restriction. All families followed this restriction for a period ranging from one to three months.

"I think there are restrictions about sex in the postpartum period. I was so weak after the birth. There should be no sex for 42 or 56 days" (Mother, urban family 1).

#### Limiting visitors

The traditional belief imposes a strict restriction on social activity and visitors. Most grandmothers and husbands in both areas, although stating they had no restriction on visitors, believed that there should be limited visitors during this period. The reasons for this were: it allows more time for the woman to rest and recuperate; some visitors may pass infections to mother or baby; they may disturb the baby or affect milk production. In the rural area some families believed that if people were to visit in the first three days, then they could continue to visit for the whole month. Other families followed the principle of allowing people to visit in the first three days and then visit again only after 14 days. Families said that this restriction is commonly known, and is not strictly imposed by the family.

"There are no restrictions, but I did not want her disturbed too often. As the baby is newborn and the mother is weak, they both need rest and a quiet environment. Some visitors may have an illness that we do not know about. The baby's immunity is poor and he may get this" (Husband, urban family 5).

In the urban area, there is a tradition that the mother can be visited whilst she is in hospital but not when she has gone home. Although most families did not have a clear restriction about this, many of their friends and families adhered to this belief.

Mothers seemed to be happy to receive visitors. Some wanted to have more visitors and social interaction. Others were happy with the number of visitors they received. In the urban area family members commented that visitors would improve the mother's mood.

"I like people visiting. I want to talk to people. I feel bored when I stay at home alone" (Mother, urban family 3).

Health workers promoted restricted visiting as they felt that too many visitors may increase the risk of illness in mother and baby. Traditional medicine practitioners did not express any views.

### Infant feeding

Interviews with families revealed the following sub-themes: breastfeeding is best, and giving honeysuckle to treat skin rashes.

#### Breastfeeding is best

All the families believed that breast milk was the best food for the baby. They said breast milk has enough nutrition for up to four months; promotes immunity in the baby; makes the uterus smaller; is convenient and is easily absorbed. They also noted that breastfeeding helps the relationship between the mother and baby.

Despite these widely held beliefs, only two mothers exclusively breastfed their babies, whilst the others gave milk powder or water during the first three days as they felt there was no breast milk for the baby. Many women continued to give milk powder or water during the month. The reasons for this were: the baby would be used to another food should the breast milk not be enough or when the mother went out; the milk powder has extra nutrition that may not be available in the breast milk; the husband can feed the baby; and breastfeeding for long periods will cause back ache.

"I think mixed feeding is very good and can provide balanced nutrition for the baby. The baby may lack something if he is only breastfed. Milk powder has other nutrients. But the milk powder is not as fresh or natural as breast milk" (Husband, rural family 2).

Respondents reported that all babies appeared healthy and were growing well. Traditional medicine practitioners supported exclusive breastfeeding; health workers expressed their support for exclusive breastfeeding, but also said that if a woman did not have enough milk it was acceptable to give milk powder. Health workers did not identify any differences in growth, occurrence of infections or jaundice between breastfed and milk powder fed babies. However, they did report that more babies who were fed milk powder had diarrhoea.

#### Giving honeysuckle for skin rash

When asked about childcare practices during the postpartum period, respondents sometimes reported giving honeysuckle as an oral medicine to the baby to relieve heat, skin rashes and eye discharge. It was related to restoring the balance of *yin *and *yang *in the baby. In the urban area four of the families used honeysuckle, whereas only one family in the rural area used this herb. Most felt that the herb was effective, but some were concerned about its effects on the baby's stomach and gut.

"We also gave some honeysuckle because the baby was hot and had some eczema. But we did not let the baby have much. I am not sure it worked, but I liked to try. I was also afraid that the herb may hurt the baby's stomach. But if we did not give this herb we would worry about the eczema" (Husband, urban family 4).

Both health workers and traditional medicine practitioners advised against this practice. They said that honeysuckle is "cold" and will harm the *yang *of the baby and damage the spleen and stomach.

## Discussion

### Evaluating the individual practices

When matched against Western standards, the individual beliefs and practices fell into three categories: some beliefs were practised and were either beneficial or had no effect on the health of mother and baby; some beliefs were adapted and were either beneficial or had no effect; and a few beliefs were followed and were harmful. Table 3 summarises the evaluation of the families' beliefs and practices.

**Table 3:** Families' beliefs and practices compared with Western health model

**Traditional model-beliefs and practice**			**Western health model: effects of reported behaviour on health, and basis for this judgement**	
**Themes**	**Beliefs**	**What is reported to be actually happening**	**Effects of actual behaviour on health**	**Explanation of these effects**

Dietary precautions	Eating more food	All consumed more food than usual.	Beneficial [23]	Provides adequate nutrients for lactation.
	Eating hot food (protein rich)	All consumed these foods.	Beneficial^[21,22,23]^	Provides adequate nutrients for lactation. Provides nutrients that promote healing of tears and incisions.
	Avoiding cold food (fruit and vegetables)	All ate vegetables, but less amount and fewer kinds.	No obvious health effects	If very restricted, could cause constipation. However, women did not report constipation as a problem.
Hygiene	No bathing or washing hair	Rural: Most bathed. Urban: Most used dry or damp towel to clean skin. Half washed hair.	No obvious health effects	
	Vulval and perineal hygiene is important	All did daily vulval and perineal hygiene.	Beneficial [24,25]	Promotes healing of tears and incisions, prevents infections.
	No brushing teeth.	Most did not practice pre-birth dental hygiene habits.	Harmful [31,32]	Allows a build up of plaque and bacteria that can cause teeth decay.
Behavioural precautions	Staying inside the home	All stayed in their homes.	No obvious health effects	
	Avoiding housework	Most did no housework.	Beneficial	Allows time to recover from childbirth and focus on caring for the baby.
	Resting in bed.	Women walked around room or home. Few did other exercises.	No obvious health effects	With complete bed rest, deep vein thrombosis is a risk but no women reported this.
	Abstaining from sex	All followed this restriction.	No obvious health effects	Allows time for the reproductive tract tissues to recover and heal.
	Limiting visitors	Most families had fewer visitors.	No obvious health effects	
Infant feeding	Breastfeeding is best	All breastfed, but only two exclusively. Most gave supplements in first 3 days and continued for whole period.	Harmful [37-40]	Interferes with milk production and breastfeeding technique, possible source of infection.
	Giving honeysuckle for skin rash	Rural: infrequently. Urban: common.	Unknown	

Some practices that are probably beneficial from a health and social perspective were eating protein rich food and larger amounts of food; family care and support; rest and focusing on recovery and the baby; and vulval hygiene.

#### a) Food

Women reported that they ate a lot of high protein or "hot" foods. Adequate protein intake aids proper wound healing and helps lactating women [21,22]. Most mothers reported more food intake than usual. This practice is consistent with WHO guidelines which recommend an increase of 10 to 20% caloric intake throughout lactation [23].

#### b) Vulval and perineal hygiene

Women in this study carried out daily vulval and perineal hygiene. This is a beneficial practice that probably helps wound healing and prevents infection [24,25].

#### c) Resting at home

Staying at home with few visitors could isolate women. On the other hand most women seemed to be mentally prepared for these thirty days and the restrictions may actually provide a supportive environment for the new mother, where she can rest, focus on looking after the baby and start to make the transition to motherhood. Some studies argue that this very situation will help protect against postnatal depression [12,26-28].

Women adapted some practices and these are either beneficial or probably have no effect on health. They include restricted bathing and eating fruit and vegetables.

#### a) Bathing

Local health managers were concerned about the harmful effects caused by women not bathing during the postpartum period. However, findings from this study show that all women carried out some hygiene practices and did not show signs of illness caused by different hygiene habits. Other studies revealed similar behaviour [9,11,12,29].

#### b) Eating fruit and vegetables

From a western health perspective, eating fewer fruit and vegetables may potentially deprive women of vitamins and contribute to constipation. WHO has a worldwide "5 a day" fruit and vegetables promotion to prevent non-communicable diseases [30]. It seems unlikely that one month of restriction would have too much effect on long-term health, although constipation may be a problem. However women were able to eat enough "warmer" fruit and vegetables to avoid this.

Some practices that are probably harmful from a western health perspective include women's limited dental hygiene, supplementary feeding of breastfed infants and giving honeysuckle to the infant.

#### a) Restricted dental hygiene

As the effects of pregnancy hormones gradually regress following delivery, dental hygiene can be beneficial in preventing dental problems during the postpartum period [31,32]. It is difficult to estimate how much damage lack of hygiene for a one-month period could have. Diet, usual dental hygiene practices and pre-pregnancy dental condition would also have an impact. Nevertheless, not brushing the teeth during this period is probably a harmful practice and dental hygiene should be promoted at all times including during the postpartum period. However, most women have moved away from the traditional practice of not brushing the teeth for the whole month.

#### b) Supplementary feeding of breastfed infants

The common practice of giving milk powder or water during the first three days and continuing to supplement breastfeeding with milk powder throughout the postpartum period is in keeping with other studies in China and with Akre's observations of hospitals worldwide [33-35]. As with Tarrant et al's study in Hong Kong the main reason for supplementing breastfeeding was a perception of insufficient milk [36]. This is a harmful practice for several reasons including the increased risk of infection in the infant and interference with breastfeeding technique and production of milk [37-40]. WHO recommends exclusive breastfeeding for the first six months of life [41]. There may be a lack of understanding about colostrum, neonate's fat stores, breastfeeding and production of milk amongst women and people influencing their decisions, including health workers. Skilled help from health workers and support from family and friends is known to influence the rate and duration of exclusive breastfeeding [29,36,42,43].

#### c) Giving honeysuckle to the infant

Finally, some families gave honeysuckle to the baby. Newborn babies commonly have skin rashes which often resolve on their own. It is not known what effect the herbs have on the alimentary canal and preparation of the herbs may cause infection. Until more evidence is available about the use of oral honeysuckle in neonates, this practice should be discouraged.

It is important to not only explore the separate practices that are carried out but also look at the ritual as a whole. All the families in this study followed the ritual of "doing the month" with some practices being adapted and others remaining fixed in tradition. These findings generate two questions: why do women follow the ritual and why do they adapt many of the practices.

### Reasons for adherence to the ritual

Several factors have been identified in this study that influence why women still follow the specific rituals given that they can exercise some degree of choice. This research supports the view that cultural rituals are important in childbearing [1-3]. It is a traditional ritual with health, social and cultural significance that supports a woman in her transition to motherhood. Whereas childbirth physically completes the process of childbearing, women observe rituals during their passage to motherhood to ensure the smooth transition from one social status to another [44]. This is also seen in other cultures such as, Thailand, Bangladesh and Zambia [5-7].

Adherence to practices is also based on past experience of significant others, most frequently the grandmother or grandmother-in law. How they did the month and how it affected their health is paramount to how they advise the new mother. Some older people experienced health problems which they related to their behaviour whilst "doing the month". They feared that similar mistakes would cause health problems for their daughters. Other older people did not experience problems, despite not strictly following the traditional practices. They tended to be more lenient about the practices and allowed their daughters more freedom. Fear of blame from family members and the wider community when problems arise, heavily influenced women's adoption of traditional practices. This relates to the important Chinese value of conforming to societal values [45].

There also was an overwhelming stoicism amongst women, that as "doing the month" only lasted for 30 days, any difficulties and inconveniences could be tolerated. Although, they all experienced some degree of frustration they all felt that the ritual was beneficial for long-term health.

### Reasons for adaptation of practices

Although women follow the ritual of "doing the month", they do adapt some of the practices. Several factors influence this modification. There is considerable western influence through the media and health professionals which can cause some conflict between more traditional beliefs of older generations and more modern beliefs of new parents. All women delivered in hospitals and so received care from health professionals who are trained in western style medicine. This care and advice will most likely influence subsequent behaviour. This has also been identified in other studies [9]. In many families in Fuqing County, members work overseas [17]. Their different experiences of health care and beliefs may have some impact on the way families in this area "do the month".

The degree to which this adaptation of practices happens will depend very much on decision-making and gender roles within the family. Chinese society is grounded in respect towards elders and this is shown in their acceptance of elders' decisions and influence [45]. Women may find it difficult to go against beliefs of their elders, without support of their husbands [44].

Some participants expressed the view that practices were developed many years ago when the situation for women was far different from today. This was also discussed in Pillsbury's study [12]. The strict ritual of "doing the month" was designed to help a woman during this difficult time. The social, economic, educational and communication developments that have taken place over the last two decades have all played a part in improving the situation for many women and generally for women of this study. Although there has been a surprising continuity of the practices of *zuo yuezi*, participants identified these socio-economic changes as important influences on the adaptation of these practices.

## Conclusion

Many of the practices carried out in "doing the month" were adapted and when they were examined individually most fell into the categories of having benefit and no effect on health. However, the important harmful practice of supplementary feeding of breastfed infants was highlighted. Further research will help delineate the extent of this practice, assess whether there are risks to health in these social contexts and explore the knowledge base of health practitioners towards breastfeeding. The study also reveals that "doing the month" continues to be an important ritual in the lives of these families. Although they wished to adhere to tradition and follow the advice of elders, some practices were adapted to the socio-economic context. These findings may help health workers utilise and build on traditional beliefs to promote health in the postpartum period as well as provide information to discourage potentially harmful beliefs.

## Competing interests

The author(s) declare that they have no competing interests.

## Authors' contributions

JR formulated the study design, carried out data collection, data analysis and wrote the manuscript. QC participated in the study design, data collection, data analysis and commented on the draft manuscript. RJT contributed to the interpretation of data and writing. PG contributed to the design, analysis and interpretation of data and writing. All authors read and approved the final manuscript.

## Pre-publication history

The pre-publication history for this paper can be accessed here:



## References

[B1] Steinberg S (1996). Childbearing research: a transcultural review. Soc Sci Med.

[B2] Lauderdale J, Andrews M, Boyle J (1999). Childbearing and transcultural nursing care issues. Transcultural Concepts in Nursing Care.

[B3] Pillsbury B, Brownlee A, Timyan J (1990). Understanding and evaluating traditional practices: a guide for improving maternal care.

[B4] Kim-Godwin YS (2003). Beliefs and practices among non-western cultures. MCN Am J Matern Child Nurs.

[B5] Kaeswam P, Moyle V, Creedy D (2003). Traditional postpartum practices among Thai women. J Adv Nurs.

[B6] Goodburn EA, Gazi R, Chowdhury M (1995). Beliefs and practices regarding delivery and postpartum maternal morbidity in rural Bangladesh. Stud Fam Plann.

[B7] Maimbolwa M, Yamba B, Diwan V, Ransjo-Arvidson AB (2003). Cultural childbirth practices and beliefs in Zambia. J Adv Nurs.

[B8] O'Dempsey TJ (1988). Traditional belief and practice among the Pokot people of Kenya with particular reference to mother and child health: 2. Mother and Child Health. Ann Trop Paediatr.

[B9] Holroyd E, Katie FKL, Chun LS, Ha HW (1997). Doing the month: an exploration of postpartum practices in Chinese women. Health Care for Women Intl.

[B10] Chen Y (2001). Chinese values, health and nursing. J Adv Nurs.

[B11] Kartchner R, Callister LC (2003). Giving Birth: voices of Chinese women. Journal of Holist Nurs.

[B12] Pillsbury B (1978). "Doing the month": confinement and convalescence of Chinese women after childbirth. Soc Sci Med.

[B13] Cheung NF (1996). Diet therapy in the postnatal period from a Chinese perspective. RCM Midwives.

[B14] Hung P (2001). Traditional Chinese customs and practices for the postnatal care of Chinese mothers. Complement Ther Nurs Midwifery.

[B15] China Statistical Publishing House (1950). Fujian Province Statistical Year Books Beijing.

[B16] Fujian province data. http://www.unescap.org/esid/psis/population/database/chinadata/fujian.htm.

[B17] Liang Z, Morooka H (2004). Recent trends of emigration from China 1982 – 2000. International Migration.

[B18] Ritchie J, Lewis J, Elam G, Ritchie J, Lewis J (2003). Designing and selecting samples. Qualitative Research Practice: A guide for Social Science Students and Researchers.

[B19] Britten N (1995). Qualitative interviews in medical research. BMJ.

[B20] Ritchie J, Spencer L, O'Connor W, Ritchie J, Lewis J (2003). Carrying out qualitative analysis. Qualitative Research Practice: A guide for Social Science Students and Researchers.

[B21] Mackay D, Miller A (2004). Wound Healing. Altern Med Rev.

[B22] Russell L (2001). The importance of patients' nutritional status in wound healing. Br J Nurs.

[B23] World Health Organisation (1998). Postpartum care of the mother and newborn: a practical guide Geneva.

[B24] Enkin M, Kierse MJNC, Neilson J, Crowther C, Duley L, Hodnett E, Hofmeyer J (2000). Perineal pain and discomfort. A guide to effective care in pregnancy and childbirth.

[B25] Chiarelli P, Cockburn J (1999). Postpartum perineal management and best practices. Aust Coll Midwives Inc J.

[B26] Heh SS, Coombes L, Bartlett H (2004). The association between depressive symptoms and social support in Taiwanese women during the month. Int J Nurs Stud.

[B27] Hung CH, Chung HH (2001). The effects of postpartum stress and social support on postpartum women's health status. J Adv Nurs.

[B28] Huang YC, Mathers N (2001). Postnatal depression – biological or cultural? A comparative study of postnatal women in the UK and Taiwan. J Adv Nurs.

[B29] Fok D (1996). Cross cultural practice and its influence on breastfeeding – the Chinese culture. Breastfeed Rev.

[B30] Fruit, vegetables and non-communicable disease prevention. http://www.who.int/dietphysicalactivity/publications/facts/fruit/en/.

[B31] Beckmann CRB, Ling FW, Laube DW, Smith RB, Barzansky BM, Herbert WNP (2002). Maternal and fetal physiology. Obstetrics and Gynaecology.

[B32] Wilkins EM (1994). The pregnant patient and the infant/toddler. Clinical practice of the dental hygienist.

[B33] Zhao Y, Niu AM, Xu GF, Garrett MJ, Greiner T (2001). Early infant feeding practices in Jinan City, Shandong Province, China. Asia and Pacific Journal of Clinical Nutrition.

[B34] Li L, Li S, Ali M, Ushijima H (2003). Feeding practice of infants and their correlates in urban areas of Beijing, China. Pediatr Int.

[B35] Akre J (1989). Infant feeding. Bull World Health Organ.

[B36] Tarrant M, Dodgson JE, Tsang Fei S (2002). Initiating and sustaining breastfeeding in Hong Kong: contextual influences on new mothers' experiences. Nurs Health Sci.

[B37] Feacham RG, Koblinsky MA (1984). Interventions for the control of diarrhoeal disease among young children: promotion of breastfeeding. Bull World Health Organ.

[B38] Habicht JP, Davanzo J, Butz WP (1986). Does breastfeeding save lives, or are appeared benefits due to biases?. Am J Epidemiol.

[B39] World Health Organisation (1998). Evidence for Ten Steps to Successful Breastfeeding Geneva.

[B40] Enkin M, Kierse MJNC, Neilson J, Crowther C, Duley L, Hodnett E, Hofmeyer J (2000). Breastfeeding. A guide to effective care in pregnancy and childbirth.

[B41] World Health Organisation (2002). The optimal duration of exclusive breastfeeding: Report of an expert consultation Geneva.

[B42] Sikorsky J, Renfrew MJ, Pindoria S, Wade A (2002). Support for breastfeeding mothers. The Cochrane Database of Systematic Reviews.

[B43] Hung BKM, Ling L, Ong SG (1985). Sources of influence on infant feeding practices in Hong Kong. Soc Sci Med.

[B44] Cheung NF (2002). Cultural and social meanings of childbearing for Chinese and Scottish women in Scotland. Midwifery.

[B45] Chinese refugee health – immigrant health. http://www3.baylor.edu/~Charles_Kemp/chinese.htm.

